# Natural coniferous resin lacquer in treatment of toenail onychomycosis: an observational study

**DOI:** 10.1111/myc.12019

**Published:** 2013-05

**Authors:** Pentti Sipponen, Arno Sipponen, Jouni Lohi, Marjo Soini, Riikka Tapanainen, Janne J Jokinen

**Affiliations:** 1 Repolar Ltd.Espoo; 2 Department of Orthopaedics and Traumatology, Päijät-Häme Central HospitalLahti; 3 Department of Cardiothoracic Surgery, Helsinki University HospitalHelsinki, Finland

**Keywords:** Resin, onychomycosis, dermatophytes, *Trichophyton rubrum*, *Trichophyton mentagrophytes*, antifungal agent

## Abstract

In *in vitro* tests, natural coniferous resin from the Norway spruce (*Picea abies*) is strongly antifungal. In this observational study, we tested the clinical effectiveness of a lacquer composed of spruce resin for topical treatment of onychomycosis. Thirty-seven patients with clinical diagnosis of onychomycosis were enrolled into the study. All patients used topical resin lacquer treatment daily for 9 months. A mycological culture and potassium hydroxide (KOH) stain were done from nail samples in the beginning and in the end of the study. Treatment was considered effective, if a mycological culture was negative and there was an apparent clinical cure. At study entry, 20 patients (20/37; 54%; 95% CI: 38–70) had a positive mycological culture and/or positive KOH stain for dermatophytes. At study end, the result of 13 patients was negative (13/19; 68%; 95% CI: 48–89). In one case (1/14; 7%; 95% CI: 0–21) the mycological culture was initially negative, but it turned positive during the study period. By 14 compliant patients (14/32; 44%; 95% CI: 27–61), resin lacquer treatment was considered clinically effective: complete healing took place in three cases (9%) and partial healing in 11 cases (85%). The results indicate some evidence of clinical efficacy of the natural coniferous resin used for topical treatment of onychomycosis.

## Introduction

Treatment of onychomycosis is a veritable clinical challenge. Oral medication with terbinafine, azoles and griseofulvin is effective and results in mycological cure among 20% to 100% of patients,^1^–^7^ but the treatment time lasts for months, is fraught with well-known risks of toxicity and relatively high costs. Successful treatment is often followed by frustrating relapses.^8^–^10^

Topical treatment options including various liquids, lacquers or devices, are used to treat mild or moderate onychomycosis and to prevent re-infections.^11^–^14^ In general practice, more than half of all patients with onychomycosis are treated with topical preparations.^15^

The settings and results of studies on topical solutions and lacquers to treat onychomycosis are heterogeneous, which makes summarising them difficult, since there are many variable treatment regimens, different patient selection criteria and major inconsistencies among the diagnostic criteria, if any. The most effective antifungal topical lacquers (e.g., 5% amorolfine and 8% ciclopirox) are considered to result in an overall rate of cure of around 30%. The cure rate is dependent on the duration of the disease, on the extent of the infection, on the involvement of the nail matrix, on trimmings of the nail and on the criteria of the subjective assessments of the final cure.^16^–^19^

In 2007, a Cochrane meta-analysis reported that there is only sparse evidence of successful management of onychomycosis with topical agents.^20^ Amorolfine may have even substantial effect, but more studies are needed. In a recent randomised and controlled study involving patients with mild-to-moderate onychomycosis without nail matrix dystrophies, long-term (13 months) topical application of terbinafine solution, amorolfine 5% nail lacquer or vehicle did not result in any differences in the rates of complete cure of onychomycosis between the study groups, albeit terbinafine solution resulted in mycological cure more often than the vehicle alone.^21^ The healing rates of onychomycosis (mycological cure or on clinical cure) with topical agents or natural products, varies from 28% to 100%, according to some recent reports.^22^–^27^

According to *in vitro* tests, resin from the natural coniferous Norway spruce (*Picea abies*) is strongly antimicrobial against a wide spectrum of microbes.^28^,^29^ The salves and lacquers composed of purified resin from spruce are antifungal, particularly against dermatophytes in agar diffusion tests. The antifungal activity is maximal when the resin content (w/w) in salve or lacquer is 30% or more.^30^ In clinical trials on skin wounds and ulcers, the coniferous resin tends to enhance the healing of wounds and wound infections.^31^–^33^ No trials or scientific reports on the clinical efficacy of coniferous resin in the treatment of onychomycosis or fungal skin diseases are available in spite of the fact that home-made coniferous resin salves have been used for centuries as a traditional remedy for various skin diseases in Northern Europe.^29^

The present prospective observational clinical trial was set up to test preliminarily whether a lacquer constructed from the natural coniferous resin shows objective evidence of efficacy in the treatment of toenail onychomycosis in the clinical setting. We hypothesised that the resin lacquer may be a treatment option in clinical practice, if the use of resin results either in mycological cure (negative culture and KOH stain) or in a positive clinical response in more than half of patients with onychomycosis who use the preparation for 9 months.

## Patients and methods

The present investigation is a preliminary observational and prospective clinical trial to test whether the topical treatment of onychomycosis with coniferous resin lacquer results in healing of fungal nail infection if the resin lacquer is applied daily for 9 months in a randomly selected group of outpatient with a clinically probable diagnosis of onychomycosis. Thus, the mycological cure and clinical cure were the primary outcome measures. The study protocol was approved and registered by the Ethics Committee of the Helsinki University Hospital, Helsinki, Finland. All patients were informed of the study course orally and by a written information sheet. Written informed consent was obtained from all patients.

The patients were recruited with an advertisement in the local newspaper in November 2010 addressing the population in the cities of Lahti and Vääksy, Finland. The advertisement informed that 40 adult volunteers with probable onychomycosis will be enrolled into the study where the clinical efficacy of a resin lacquer to treat onychomycosis is tested. In the days following the announcement, more than 120 patients signed up at the local medical clinic (Vääksy Medical Centre, Vääksy, Finland). The first 40 consecutive patients were accepted without exclusions. Clinical examinations by three physicians (AS, consultant orthopaedic surgeon; JJ, consultant cardiothoracic surgeon; PS, professor of pathology) were arranged and 37 patients with clinically probable onychomycosis were enrolled into the final study population. During the study period, all patients used only resin lacquer to treat their onychomycosis. The classification of onychomycosis was determined according to Baran *et al.*^34^ Demographics, disease history, disease severity and classification of onychomycosis in the study population are presented in Table 1.

**Table 1:** Patients’ demographics, disease history and data on extent and clinical type of onychomycosis

Parameter (*n* = 14)	All patients (*n* = 33)	Mycology + at study entry (*n* = 19)^1^	Mycology − at study entry (*n* = 14)^1^
Demographics			
Male/female	17 (52)/16 (48)	13 (68)/6 (32)	4 (29)/10 (71)
Age (years)	63 ± 12 [36–80]	65 ± 14 [37–80]	60 ± 8 [36–72]
Disease history			
<5 years	6 (18)	2 (10.5)	4 (29)
5–10 years	5 (15)	2 (10.5)	3 (21)
>10 years	22 (67)	15 (79)	7 (50)
Extent of onychomycosis			
Toenails affected	33 (100)	19 (100)	14 (100)
Big toe affected	33 (100)	19 (100)	14 (100)
Severity of onychomycosis			
Mild	15 (45)	7 (37)	8 (57)
Severe	18 (55)	12 (63)	6 (43)
Clinical type of onychomycosis			
WSO	3 (9)	0 (0)	3 (21)
DLSO	22 (67)	13 (68)	9 (64)
TDO	8 (24)	6 (32)	2 (14)
Clinical treatment history			
Previous oral antifungal therapy	7 (21)	5 (26)	2 (14)
Previous topical therapy	10 (30)	4 (21)	6 (43)
Clinical cure among compliant patients			
Data not available	1 (3)	1 (5)	0 (0)
No improvement	18 (55)	12 (63)	6 (43)
Improvement	11 (33)	6 (32)	5 (36)
Complete clinical cure	3 (9)	0 (0)	3 (21)

Values denote number of patients (percentage) or mean ± standard deviation [range]. WSO, white superficial onychomycosis; DLSO, distal and lateral subungual onychomycosis; TDO, total dystrophic onychomycosis.

1 Mycology + or − denotes mycological culture or potassium hydroxide (KOH) stain positive or negative.

**Figure 1:**
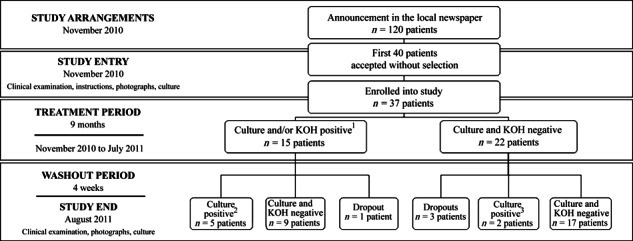
Flow chart of the trial: data of the patients who had positive or negative mycological culture in the beginning of the study. ^1^*Trichophyton rubrum* in 11 cases and *Trichophyton mentagrophytes* in four cases. ^2^*Trichophyton rubrum* in three cases and *Trichophyton mentagrophytes* in two cases. ^3^*Trichophyton rubrum* in one case and *Trichophyton mentagrophytes* in one case.

The study started in November 2010 and ended in August 2011. In the beginning of the study, the physicians (AS, JJ, PS) informed the patients about the biology of onychomycosis and of the necessity of a long-lasting treatment. Mycology samples for culture and digital photographs were taken from the most severely affected nail at the beginning and the end of the study. At the end of the study, all patients were ordered to discontinue using the resin lacquer 4 weeks before the last visit (washout period). During that visit, the patients underwent a final clinical examination and samples were collected for culture and KOH stain. During the trial, all participants were contacted at least twice by phone to monitor the patient’s compliance and willingness to continue in the trial. Four of the 37 patients (11%) dropped out before the conclusion of the study (Fig. 1).

Samples for mycological cultures and KOH microscopy were obtained at study entry (before the start of the treatment) and at study end (after a 4-week washout period). Nail clippings or curettage samples were taken by physicians from the most affected areas of the nail and cultured for mycology according to instructions of the mycological laboratory. Cultures and KOH microscopy were done in an independent specialised mycology laboratory with standard techniques (Medix Ltd., Helsinki, Finland).

### Mycological and clinical cure

1. Mycological cure was defined as a negative culture and KOH stain at the conclusion of the study in cases where initial culture and/or KOH of the sample had been positive.
2. Partial clinical cure was defined as a normal and healthy nail matrix in areas of the nail which had been affected at study entry.
3. Complete clinical cure was defined as a negative culture result and a fully normal and healthy nail at study end.

The level of clinical cure was assessed by the three physicians (AS, JJJ, PS), and it was based on analysis of digital photographs of the most severely affected nails taken at the beginning and the end of the study.

### Coniferous resin lacquer

The lacquer (Abicin® Resin Lacquer 30%; Repolar Oy, Espoo, Finland) was constructed and manufactured from resin of the natural coniferous Norway spruce (*Picea abies*) originating from the Finnish Lapland. The resin content (w/w) of the lacquer was 30%. The other ingredients were monopropylene glycol, glycerol and alcohol.

The lacquer was given to patients for self-medication in 10 ml bottles equipped with an applicator brush. The patients were asked to apply the solution daily on the affected nails. The solution dries in 30 min and forms a soft spotless cover on the nail. Before application, the patients were advised to wash and bathe the nail in warm water, and then to file and cut the nail. If possible, the patients were advised to visit a podiatrist for nail trimming.

### Statistics

Data were analysed and reported according to the STROBE guidelines.^35^ Qualitative data are expressed as frequencies and percentages and quantitative data as mean ± standard deviation. Evidence of clinical efficacy would be assessed with a study population of 40 patients. This calculation was based on power analysis and assumption that half (*n* = 20) of the patients would have culture-positive onychomycosis at study entry. If 10 of these 20 patients would obtain mycological cure, the mean mycological cure rate would be 50% and the lower 95% confidence limit of the cure rate would be more than 25%, indicating a positive treatment effect. SPSS version 17.0 was used as the statistical software (IBM Corp, Armonk, NY, USA).

## Results

**Figure 2:**
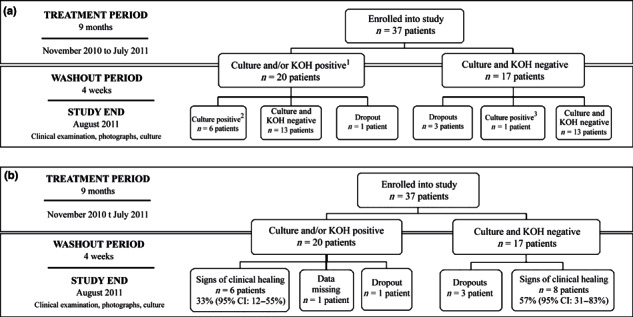
(a) Data of the patients who had positive and negative mycology (culture and/or KOH stain) in the beginning of the study. ^1^*Trichophyton rubrum* in 11 cases and *Trichophyton mentagrophytes* in four cases. ^2^*Trichophyton rubrum* in four cases and *Trichophyton mentagrophytes* in two cases. ^3^*Trichophyton mentagrophytes* in one case. (b) Complete and partial healing rate among the 33 compliant patients. One patient was dropped due to missing photograph at the end of the study.

The results of mycological cure are presented in Figs 1 and 2. Of 14 eligible, culture-positive patients at study entry, 9 patients (65%; 95% CI: 42–87) turned culture- and KOH stain-negative by study end (Fig. 1). Among 14 compliant culture- and KOH stain-negative patients at study entry, one patient (7%; 95% CI: 0–21) turned culture-positive (*Trichophyton mentagrophytes*) at study end (Fig. 2a). Thirteen of 19 eligible and compliant patients (68%; 95% CI: 49–89) of 20 patients who had been culture-positive for dermatophytes and/or positive for KOH staining at study entry, were culture- and KOH stain-negative at study end (Fig. 2a). Four of eight eligible patients with both positive KOH stain and positive culture at study entry turned culture-negative at study end (50%; 95% CI 15–85).

**Figure 3:**
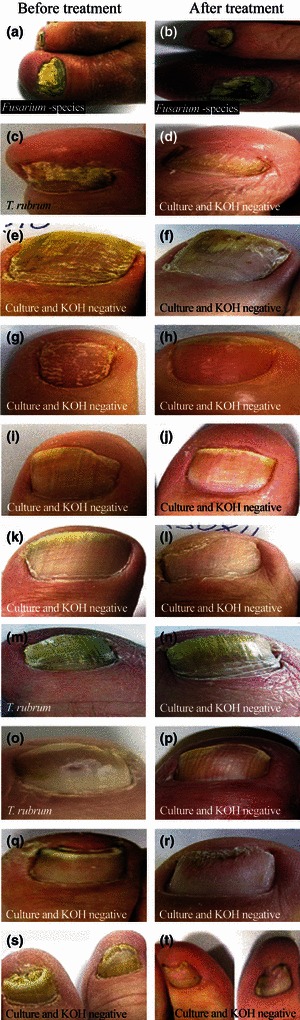
Photographs, mycological culture, KOH stain and the treatment result of 10 patients before (left panel) and after (right panel) resin lacquer treatment (a–t). Non-healed TDO (a, b) and partially healed DLSO (c, d). Partially healed WSO (e, f) and completely healed WSO (g, h). Two completely healed DLSO (i–l). Two partially healed DLSO (m–p). Two partially healed DLSO (q–t).

Assessments of the clinical cure of onychomycosis among the initially culture-positive and culture-negative patients at study entry are summarised in Table 1 and Fig. 2b. Examples of cases with non-healed, partially healed and completely healed onychomycosis are presented in Fig. 3. Complete cure was recorded in three cases, all of which occurred in patients with negative mycology (negative KOH stain and negative culture) at study entry (Fig. 3g–l). In one of these patients, onychomycosis was classified as white superficial onychomycosis (WSO; Fig. 3g,h), and the two others as distal and lateral subungual (DLSO) type of onychomycosis (Fig. 3i–l). Altogether, a trend towards clinical cure (complete or partial) was observed in 14 of 32 eligible patients (44%; 95% CI: 27–61). Four of eight patients (50%; 95% CI: 15–85) who had been culture-positive at study start and were culture-negative at study end had partial healing of their onychomycosis by clinical assessment. One of six patients (17%; 95% CI: 0–46) with clinical signs of healing were mycology positive both at study start and at study end. There were no allergic reactions or other adverse events among the study population during the study.

## Discussion

To our knowledge, this is the first clinical study on the efficacy of a natural coniferous resin in the treatment of onychomycosis. It is known that the natural coniferous resin from the Norway spruce (*Picea abies*) has a strong antimicrobial activity and that the resin is strongly antifungal against all dermatophytes causing onychomycosis in man.^28^–^31^

This study is preliminary and observational, but it suggests that topical long-term use of coniferous resin, in the form of lacquer at a concentration of 30%, may have a beneficial effect on the healing of onychomycosis. This positive treatment effect was implied by the fact that more than 60% of the cases where dermatophytes had been cultured the start of the study had turned culture-negative by the end of the study. There was also clinical improvement in 40% of the patients, and three patients underwent complete cure of their onychomycosis. These cure rate are comparable to those obtained with ciclopirox, fluconazole and some other topical agents.^19^,^22^,^24^–^27^,^36^

Mycological cure and clinical cure of onychomycosis were the primary outcome measures of treatment efficacy. Neither of these measures is ideal. It has been reported that placebo treatment may turn a culture-positive onychomycotic nail culture-negative in up to 50% of patients.^36^ In addition, the techniques in nail tissue sampling have a strong impact on the accuracy and outcome of the fungal culture and may result in false negative results.^37^ In the present trial, the positive mycological response rate was clearly higher than 50% suggesting that at least a temporary treatment response against dermatophytes was achieved in some, but not all, patients, and that the positive response rate was not fully biased. This conclusion is supported by the observation that only one patient was culture-positive at study end among those patients who initially were culture-negative.

Assessment of the clinical cure of onychomycosis is highly subjective as well, and is also subject to bias. Non-fungal nail affections may resemble dermatophytic onychomycosis, and this may result in a wrong diagnosis, both at study entry and study end. Differential diagnostic alternatives are psoriasis, chronic traumatic injuries, chronic eczemas, red lichen and chronic paronychia.^38^–^40^ The possibility of such false diagnoses cannot be excluded in the present study among the culture-negative patients although the diagnosis of “probable onychomycosis” was stated by physicians at study entry. In future studies, it would be preferable to enrol only patients who have either culture, or KOH stain or PAS (periodic acid-Schiff) stain proven onychomycosis.

In the per protocol analysis, 44% of 32 eligible patients who completed 9 month trial, showed subjective signs of a positive clinical treatment effect of coniferous resin (one patient was dropped due to the absence of photographs). A positive treatment effect was, however, also seen in 57% of the patients who had no objective mycological evidence (negative culture and KOH stain) of dermatophytic onychomycosis. Coniferous resin has, in addition to its antifungal activity, anti-inflammatory property and a strong antimicrobial activity against a wide range of bacteria. These effects are related to resin acids, lignans and coumaric acids present in natural coniferous resin.^29^,^31^ Therefore, any bacterial nail infections, mixed infections, paronychia, non-dermatophytic fungal infections, nail psoriasis and eczemas may respond positively to the natural coniferous resin.

In general, half of the patients with clinically probable onychomycosis are reported to be negative for pathogenic fungi by conventional mycological cultures.^39^ However, dermatophytes or pathogenic moulds can be found more frequently in samples from ill nails when more sophisticated diagnostic techniques are used.^30^–^46^ In the present study population, roughly 50% of the enrolled patients were culture-positive for dermatophytes by conventional mycological cultures at study entry.

The cure rate of onychomycosis varies remarkably between published studies. The reasons for the variable results are related to differences in patient selection, disease duration and the type and severity of the onychomycosis.^47^ In this study, the patients were not selected with regard to these or other variables. Since enrolment was based on an advertisement in the local newspaper and on initial self-diagnosis of onychomycosis, more severe and chronic cases of onychomycosis may have accumulated in this study population. A disease history of more than 10 years was reported by more than half of the patients. In addition, at least half of the patients reported that they had tried several oral or topical treatments without success over the years. Although none of the patients used any oral or topical treatment in the beginning of the study, we were not able to report potential washout period between the patients’ last treatment effort and the initiation of resin lacquer treatment. In the case of relatively short washout period, the results may be biased due to prior treatment. Also the variability in the degree of nail involvement between the patients, variability of pathogenic organisms, as well as the small number of recruited patients could be considered as potential limitations of our study. In all 37 compliant patients, the toenails, particularly the big toes, were affected, and more than one nail was affected in most of the patients. This corresponds well with the clinical appearances of onychomycosis in the general population.^48^

The onychomycosis was of the WSO or DLSO type in 25 cases (76%), and of TDO type in eight cases (24%). It seems that the WSO and DLSO types of onychomycosis are more likely to respond to the topical coniferous resin treatment than the TDO type. Only one of the eight TDO cases showed signs of partial clinical healing, suggesting that onychomycosis in severely dystrophic nail may not be an ideal target for topical treatment efforts.

In conclusion, the present trial indicates some preliminary, positive clinical efficacy in usage of natural resin as topical treatment of onychomycosis. Randomised and controlled clinical trials are needed to corroborate this claim and to provide details on which subjects benefit the most from this form of treatment of onychomycosis.
